# Fusion of permanent teeth as post-traumatic sequelae of trauma in primary dentition: A case report with fifteen years of follow-up

**DOI:** 10.4317/jced.54856

**Published:** 2018-07-01

**Authors:** Vanessa Costa, Ivam da Silva-Júnior, Ayah Shqair, Andressa Gastmann, Elaine Baldissera, Marília Goettems, Dione Torriani, Rudimar-Antonio Baldissera, Thiago-Marchi Martins

**Affiliations:** 1 PhD, School of Dentistry, Federal University of Pelotas, Pelotas-RS, Brazil; 2 MsC, Post Graduate Program in Dentistry, School of Dentistry, Federal University of Pelotas, Pelotas-RS, Brazil

## Abstract

Traumatic dental injuries in primary dentition present risk of sequelae in the permanent dentition. In this case report, we describe the management and long term follow-up of sequelae affecting permanent central incisor due to prior intrusive luxation and subluxation of the corresponding primary tooth. A 5-year-boy was referred for treatment, with history of fall by the age of 21 months, which caused subluxation of the primary maxillary right and left central incisors, and intrusion of the maxillary right lateral incisor. Radiographic and clinical monitoring was regularly performed. Hypoplasia and crown dilaceration of the permanent maxillary right central incisor was detected, as well as an enamel bridge between the central and lateral right incisors was diagnosed by cone bean tomography. Gingevectomy followed by the breaking of the enamel junction between the crowns of lateral and central incisors and indirect facet in composite resin were used to treat the sequelae. A precise diagnosis, involving a multidisciplinary team, contributed to the success of treatment.

** Key words:**Case reports, follow-up studies, pediatric dentistry, tooth, deciduos, complications.

## Introduction

Traumatic dental injuries (TDI) in primary dentition present risk of sequelae in the permanent dentition, due to the close relationship between the apices of primary teeth and the developing permanent successor buds (1). In the permanent teeth, the prevalence of developmental disturbance following injuries to their predecessors varied from 12 to 74% (2). Usually, these sequelae can affect the crown, root or the entire bud of the permanent successor. Clinically, alterations may occur such as hypoplasia of the enamel, dilaceration of the crown, and discoloration (1). On the other hand, other complications affecting the root region may be detected include: duplication and partial or total dilaceration. Other sequelae include alterations in the eruption process may be identified during the follow up process (3).

Intrusive luxation is the type of TDI most likely to cause mineralization disturbances in permanent successors, with frequencies ranging from 41 to 77% (1). Innes (4) analyzed intrusive injuries of primary incisors in children under 4 years of age and found that over half of the permanent successors were found to have one or more developmental disturbances. In this case report, we describe the management and long term follow-up of sequelae affecting permanent central incisor due to prior intrusive luxation and subluxation of the corresponding primary tooth.

## Case Report

A healthy 5 years-old boy was referred to the Centre for the Study and Treatment of Dental Trauma in Primary Dentition (NETRAD- Federal University of Pelotas, Brazil), after 3 years and 5 month of follow-up in private dental clinic. Reportedly, at 21 month of age, he had experienced a fall from the proper high, while riding his scooter that caused subluxation of the primary right and left maxillary central incisors, and partial intrusive luxation of the primary maxillary right lateral incisor.

Clinical and radiographic examination revealed crown discoloration of both primary maxillary right and left central incisors and the maintenance of the intrusion of the primary maxillary right lateral incisor. Also, mobility of the primary maxillary right central incisor and pulp necrosis of the primary maxillary left central incisor was detected. Endodontic treatment was performed in the primary maxillary left central incisor. Extraction of the primary maxillary right lateral incisor was performed, because of the lack of re-eruption. The patient was oriented to regular follow-up every 6 months.

**Figure 1:**
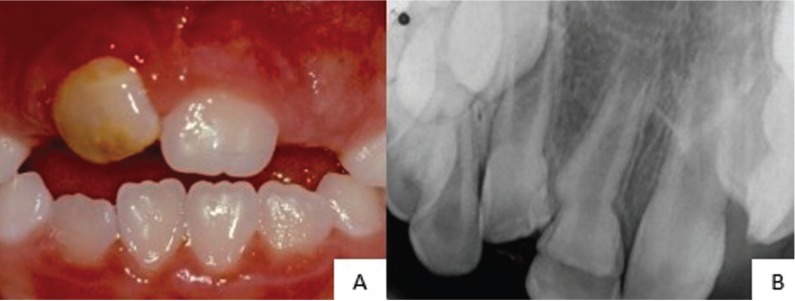
(a) Clinical presentation of the child at 8 years of age showing hypoplasia and crown dilaceration of the permanent upper right central incisor (b) Radiographic examination.

By the time the patient had eight years and 5 months of age, developmental disturbances like hypoplasia and crown dilaceration of the permanent maxillary right central incisor were confirmed, as previously observed radiographically (Fig. 1). For aesthetic reasons, restorative treatment was performed with resin composite for both labial and palatal surfaces. After 2 years, in another follow-up assessment, the absence of eruption of the permanent maxillary right lateral incisor was diagnosed and cone bean computed tomography was requested. Bond between the crowns of maxillary right central and lateral incisors through enamel bridge was observed.

A multidisciplinary treatment plan, involving periodontist, orthodontist and pediatric dentists, was outlined in an attempt to expose the teeth through gingivectomy for the orthodontic traction. During the gingivectomy procedure, an attempt was performed to display the crown of the maxillary right lateral incisor, the junction between the crowns of the maxillary right lateral and central incisor was separated using diamond bur, allowing the bracket bonding for orthodontic traction.

**Figure 2:**
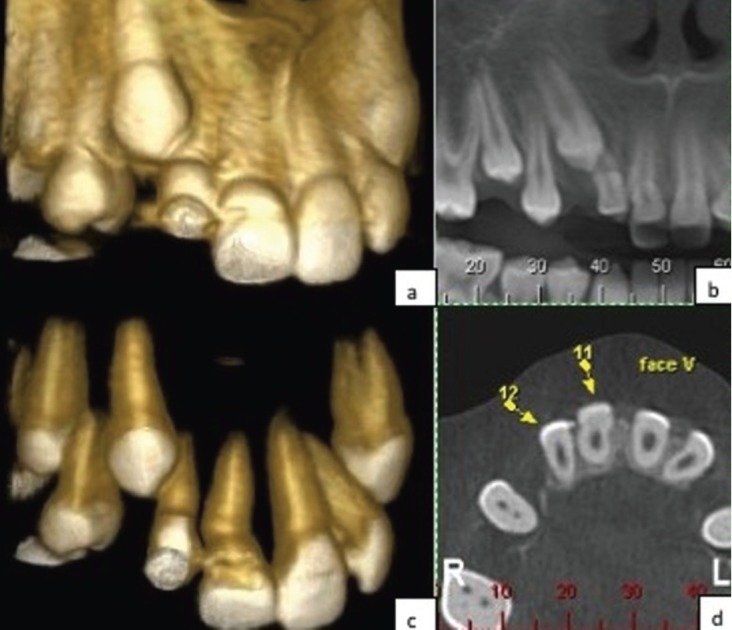
Cone beam computed tomography showing the enamel bridge between central and right lateral incisor still stayed after a first attempt of separation. (a) Vestibular view (b) Panoramic view (c) Vestibular view with removal bone tissue (d) Axial cutting.

Traction of the lateral incisor was attempted using brackets and later using cantilever systems. However, due to lack of results through the traction attempts, new cone beam CT was requested and it was noticed that the enamel bridge between central and right lateral incisor still stayed (Fig. 2). Thus, periodontal surgery was conducted and complete separation of the enamel bridge was obtained, in an attempt to allow the eruption of the maxillary right lateral incisor. Thereafter, indirect facet in composite resin was made to this element.

**Figure 3:**
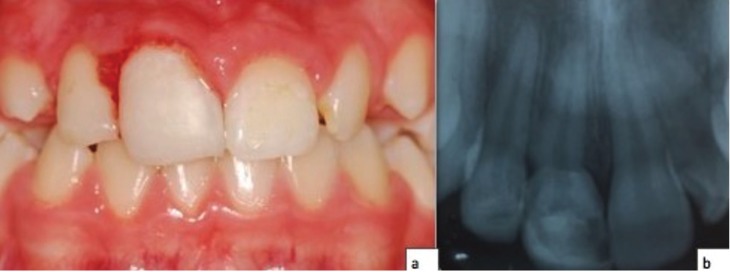
(a) Intra-oral photograph after restoration, (b) Periapical X-ray.

Currently, the patient is 16 years (Fig. 3) and continues in regular clinical and radiographic follow-up. The informed consent of the patient was obtained for this publication.

## Discussion

In the case here presented, a child suffered a severe injury in primary teeth and had sequelae in the permanent dentition, causing physical and emotional consequences to the children and the family. An analysis of records of patients attending NETRAD showed that intrusion and subluxation are the most common types of injury in this treatment center (5,6). . These injuries are frequently associated with sequelae, which can affect both the permanent successor and the traumatized tooth itself (1).

Spontaneous re-eruption is expected following intrusion in primary teeth (7). In the present case, the extraction was performed because the tooth did not re-erupt. In these cases, extraction is recommended because of the risk of damage presented to the permanent bud (8). Also, according to the most up-to-date guideline (9), the direction of the apex displacement is essential to define the treatment in cases of intrusive luxation. If the apex is displaced toward or through the labial bone plate, the tooth is left for spontaneous repositioning, however if the apex is displaced into the developing tooth germ, tooth should be extracted.

In this case, the severity of the injury, associated with the young age of the patient, caused alterations in shape and color in the permanent successor, which caused a severe function and esthetic sequelae. The germ of the permanent successor was in early stages of Odontogenesis. Probably, trauma promoted destruction of ameloblasts in the enamel epithelium, favoring the occurrence of dilaceration and hypoplasia. Histologically, the existing union between the elements can be explained by the approach and fusion of the enamel epithelium after traumatic shock, thus creating a “gate”, this began to synthesize the enamel matrix, which just then being produced in a single block, after the enamel maturation phase remains the bridge between the dental elements.

The treatment of permanent incisors crown dilaceration depends on the severity of the abnormality. It is essential to perform an accurate diagnosis in order to elect an effective conduction reestablish function, aesthetics and self-esteem of the patient. In this case, a composite resin restoration to the maxillary right central incisor, using an opaque color, was performed in order to mask the characteristic yellow-brownish color (10).

In this report, in addition to the damage generated at the maxillary right central incisor, the lateral on the same side was impacted. In these cases the most common treatment options are surgical extrusion followed by orthodontic traction (11), or extraction of the impacted element followed by prosthetic rehabilitation (12). In the present case, orthodontic traction was attempted twice, but did not achieve the expected success, probably due to the continuity of the enamel bridge. The extraction of impacted element followed by prosthetic rehabilitation was not applied in this case because the extraction of tooth in a young patient would result in a horizontal and / or vertical alveolar bone defect (13).

Correct diagnosis is the key of successful treatment. Uses of adjunct tools like radiographs are important to provide valuable information that may affect the treatment plan for the injured tooth. Panoramic examination is a very useful one, but provides only a two-dimensional picture of three-dimensional structures which may be considered one of its limitations (14). Therefore, Cone beam computed tomography (CBCT) provides multiple plans to identify with precision three-dimensional anatomical details, it has shorter exposure time, high resolution, reduced image artifact, low radiation dose and high accuracy (15). However, it must be indicated for specific situations and the high cost of this technology restricts its use in most dental offices.

The periodic follow-up examinations facilitate and expedite the provision of adequate treatment for children sustaining any sequelae in the permanent anterior teeth following trauma in their predecessors (2). In this particular case, it was possible to identify an enamel bridge formed between two dental elements, formed as a result of the traumatic injury. The patient had fifteen years of follow-up and the frequent monitoring enabled to detect the consequences of deciduous and permanent teeth and to intervene when necessary. Despite the difficulties, this type of cases can be treated with success. A precise diagnosis, preferably involving a multidisciplinary team, is of outmost importance to detect any possible alterations resulting from dental trauma.
